# Promising preclinical patient-derived organoid (PDO) and xenograft (PDX) models in upper gastrointestinal cancers: progress and challenges

**DOI:** 10.1186/s12885-023-11434-9

**Published:** 2023-12-07

**Authors:** Jing Gao, Jianqiang Lan, Haiyan Liao, Fang Yang, Pei Qiu, Feng Jin, Shubin Wang, Lin Shen, Tengfei Chao, Cheng Zhang, Yu Zhu

**Affiliations:** 1 Department of Oncology, Shenzhen Key Laboratory of Gastrointestinal Cancer Translational Research, Cancer Institute, Peking University Shenzhen Hospital, Shenzhen-Peking University- Hong Kong University of Science and Technology Medical Center, Shenzhen, China; 2 Guangdong Research Center of Organoid Engineering and Technology, No. 11 Kaiyuan Avenue, Huangpu District, Guangzhou, China; 3 Key laboratory of Carcinogenesis and Translational Research (Ministry of Education/Beijing), Department of Gastrointestinal Oncology, Peking University Cancer Hospital and Institute, No. 52 Fucheng Road, Haidian District, Beijing, China; 4 Department of Oncology, Tongji Hospital, Tongji Medical College, Huazhong University of Science and Technology, No. 1095 Jiefang Avenue, Qiaokou District, Wuhan, China

**Keywords:** Upper gastrointestinal cancer, Organoid, PDX, Personalized therapy

## Abstract

Gastrointestinal (GI) cancers (gastric cancer, oesophageal cancer, liver cancer, colorectal cancer, etc.) are the most common cancers with the highest morbidity and mortality in the world. The therapy for most GI cancers is difficult and is associated with a poor prognosis. In China, upper GI cancers, mainly gastric cancer (GC) and oesophageal cancer (EC), are very common due to Chinese people’s characteristics, and more than half of patients are diagnosed with distant metastatic or locally advanced disease. Compared to other solid cancers, such as lung cancer and breast cancer, personalized therapies, especially targeted therapy and immunotherapy, in GC and EC are relatively lacking, leading to poor prognosis. For a long time, most studies were carried out by using in vitro cancer cell lines or in vivo cell line-derived xenograft models, which are unable to reproduce the characteristics of tumours derived from patients, leading to the possible misguidance of subsequent clinical validation. The patient-derived models represented by patient-derived organoid (PDO) and xenograft (PDX) models, known for their high preservation of patient tumour features, have emerged as a very popular platform that has been widely used in numerous studies, especially in the research and development of antitumour drugs and personalized medicine. Herein, based on some of the available published literature, we review the research and application status of PDO and PDX models in GC and EC, as well as detail their future challenges and prospects, to promote their use in basic and translational studies or personalized therapy.

## Introduction

Gastrointestinal (GI) cancers (including gastric cancer, oesophageal cancer, liver cancer, colorectal cancer, and pancreatic cancer) pose a major challenge to public health and have the highest morbidity and mortality in the world [1, 2]. Upper GI cancers mainly include gastric cancer (GC) and oesophageal cancer (EC), and there were nearly 1.7 million new diagnoses and over 1 million new deaths for GC and EC combined, accounting for 8.7% and 13.2% of the incidence and mortality of all cancers in 2020 [3]. In China, GC and ESCC (oesophageal squamous cell carcinoma, a type of EC that is dominant in China) are very common in people with Chinese characteristics, and more than half of patients are diagnosed with distant metastatic or locally advanced disease and have thus missed the opportunity for radical surgery and have a very poor prognosis [4].

The standards of care for GC and EC patients mainly include surgical resection, drug therapy (chemotherapy, targeted drug therapy, immunotherapy, etc.), and radiotherapy [5–8]. For patients with advanced disease, drug-based comprehensive treatment is the main method [9]. In the era of precision medicine, along with the emergence of many new techniques represented by next-generation sequencing (NGS), significant progress has been made in increasing patient survival with the development of targeted drugs and immunotherapy drugs in a variety of tumours including GC and EC [10, 11]. However, precision therapeutic options for cancer patients are far from being met, especially for GC and EC, compared to other solid cancers, such as lung cancer and breast cancer [12]. Over the past decade, most clinical trials of targeted drugs in GC and EC have failed and produced negative results, and one of the reasons for the failure was the misdirection of preclinical results [13].

**Fig. 1:**
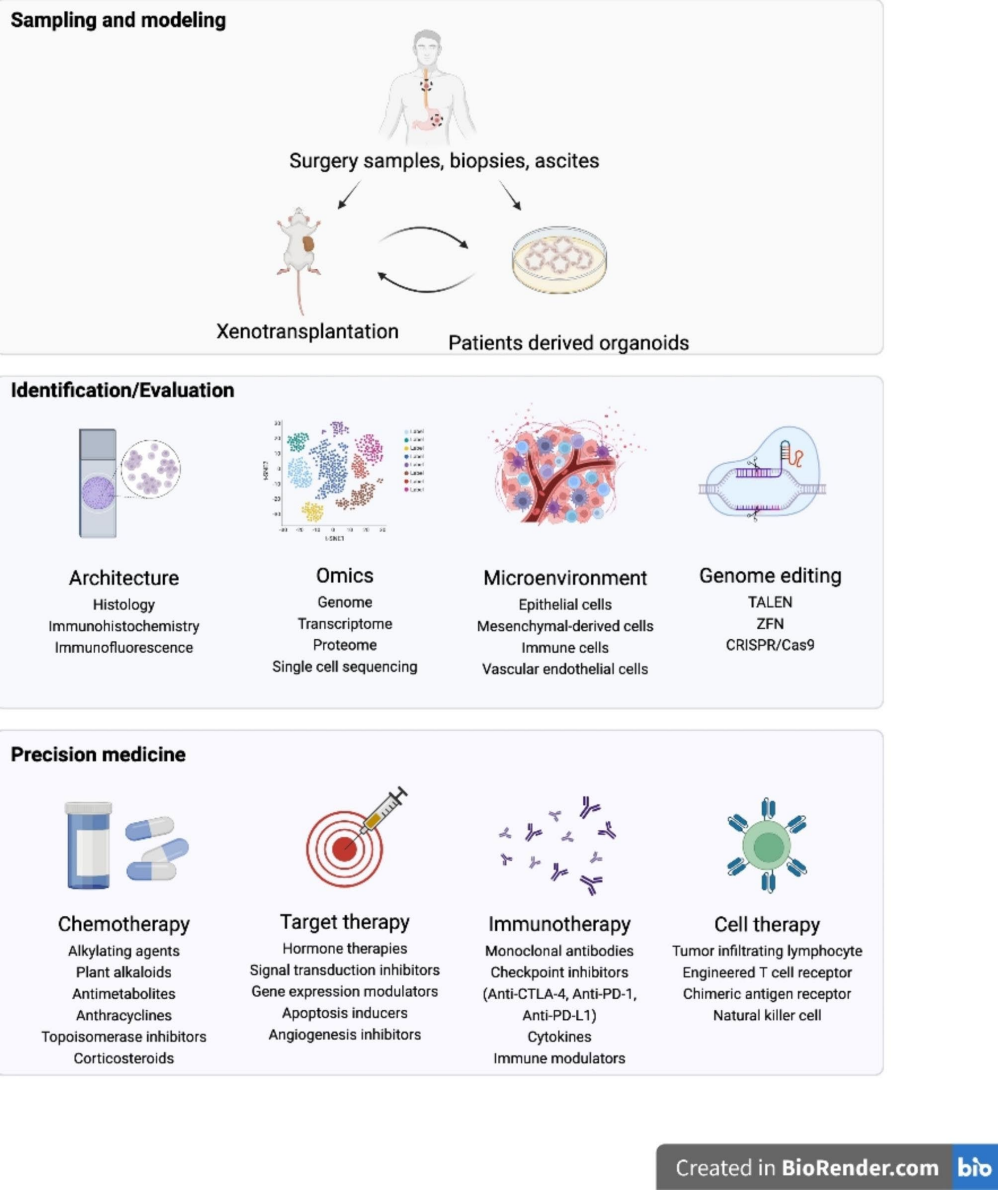
Flow diagram of organoid and PDX models derived from patients

For a long time, most preclinical studies were carried out by using in vitro cancer cell lines or in vivo cell line-derived xenograft (CDX) mouse models, which lack the genetic heterogeneity of original tumors after many passages, are unable to reproduce all the essential characteristics of tumours derived from patients [14–16]. Therefore, cell lines and CDX models have failed to predict human efficacy for most drugs, especially targeted therapeutics [17]. To generate preclinical results that are more reliable, patient-derived organoid (PDO; hereafter referred to as organoids) and xenograft (PDX) models have attracted attention in recent years [18, 19]. Either organoid (ex vivo 3D cell model) or PDX (in vivo animal model; mice are generally used) models are mainly constructed using tumour tissues derived from patients (malignant body fluids can also be used successfully) and preserve the heterogeneity and most of the features of patient tumours [20]. Although organoid and PDX models have different characteristics with advantages and disadvantages (Table 1), due to the high consistency with the features of patient tumours, both models have been widely used in numerous studies, especially in the research and development of antitumour drugs and personalized medicine (Fig. 1).

**Table 1:** The differential features of organoid and PDX models

Characteristics	Organoids	PDX model
Model type	Ex vivo	in vivo
Patient recapitulation	Yes	Yes
Stability through passage	Yes	Yes
Tumour microenvironment	Rare, few or none	Yes (from host)
Maintenance of immune response	No	No
Methods to recapitulate immune response	Coculture with immune-related cells	Introduce humanized immune system
Scalability	High	Medium
Establishment time	Relatively fast, ~weeks	Generally slow, ~months
Cost	Relatively low	High
Current applications	Preclinical study, translational research, personalized drug screening, etc.	Preclinical study, translational research, personalized drug screening, etc.

It is well known that personalized medicine guided by genotyping is the mainstream precision medicine at present [21]. In addition, personalized functional diagnosis using patient-derived models has emerged as an alternative method to guide personalized therapy [22], which is often referred to as an ‘avatar’ of a patient to predict the therapeutic efficiency of some drug or drug combination. For patients with rare defined driver genes or few actionable variants, personalized therapy guided by functional diagnosis seems to be very important. Herein, based on the available published literature, we review the research and application status of organoid and PDX models in GC and EC, as well as their future challenges and prospects, to promote their use in basic and translational studies or personalized therapy for patients.

### Organoid and PDX models in EC

#### The culture and application of organoids in EC

Based on the few published studies, organoids could be cultured from both the mucosal epithelium and tumour tissues of EC patients using suitable media contianing a variety of growth factors, inhibitors and hormones, using matrigel or basement membrane extract as ECM substitutes [23, 24]. Zheng et al. developed a novel oesophageal minimum essential organoid culture medium (E-MEOM) for culturing murine oesophageal organoids that were immunohistologically and transcriptomically similar to the normal oesophageal epithelium [25]. Vega et al. developed a 3D organotypic culture system and demonstrated that inhibition of Notch signalling promoted the transdifferentiation of normal oesophageal squamous epithelium to a Barrett’s oesophagus (BE)-like metaplasia partially through KLF4 upregulation [26]. Karakasheva et al. developed a protocol that could successfully generate primitive organoids from oesophageal squamous cell samples, allowing the study of the molecular mechanisms underlying oesophageal cancer cell propagation in PDO culture [27]. Kijima et al. established PDOs from both tumour and adjacent normal mucosa biopsies of ESCC patients using medium with exogenous stem cell factors, and the success rate of PDO culture was 68.75% (11/16) for ESCC tumour tissues [28]. They evaluated the growth and structural characteristics of ESCC PDOs treated with 5-FU ex vivo and found that treatment resistance was more conducive to tumour-like organ formation, and the potential treatment-resistant cell population was characterized by high CD44 expression and high autophagy capacity [28]. These organoid culture systems provided a comprehensive experimental platform for studying the molecular mechanisms of oesophageal cancer cell development and drug response. However, at present, the successful rate of esophageal cancer organoids is generally low, and it’s sincerely expected that further technical optimization can overcome this issue.

Organoids have been reported to maintain high accuracy in predicting therapy response in cancer patients (Table 2). Vlachogiannis et al. reported the potential of PDOs to predict therapy response using a living biobank, which included 110 fresh biopsies from 71 patients, with 100% sensitivity and 93% specificity to forecast patients’ responses to chemotherapy or targeted therapy [29]. Li et al. established long-term expansion PDOs from resection tissues of oesophageal adenocarcinoma cancer (EAC) patients using a glandular-preferred protocol which added additional Wnt3A to the growth factors combination of culture medium and tested 24 anticancer compounds in these PDOs [30]. For target agents, the drug sensitivity of PDOs was mostly in accordance with the molecular status, such as *TP53* and *EGFR* mutations. For chemotherapeutic drugs, including 5-fluorouracil, epirubicin, and cisplatin, the resistance of PDOs was consistent with the response in patients [30]. Another study reported that a total of 16 EAC PDOs were successfully grown and characterized using short tandem repeat (STR) analysis, whole-exome sequencing (WES), histology, and immunohistochemistry, indicating recapitulation of the tumour histology and genomic characteristics [31]. Additionally, drug testing using clinically appropriate chemotherapeutics and targeted therapeutics showed an overlap between the patient tumour response and the corresponding organoid response. Together with genomic features, an EAC PDO carrying ERBB2 amplification responded to the targeted HER2 agent mubritinib, while wild-type organoids showed no response, providing insight into personalized treatment using PDO-based drug sensitivity tests. Taken together, due to the capacity of PDOs to recapitulate patient responses, these results verified the feasibility of using organoids for testing drug sensitivity.

**Table 2:** Organoid culture and application in EC

Reference	Sample source	Tissue type	Sample number	Success rate of transplantation	Evaluation
[30]	EAC	Surgical resections	32	10/32 (31.25%)	H&E, immunohistochemistry, immunofluorescence, whole-exome sequencing, therapy response evaluation
[27]	ESCC	Endoscopic biopsies	16	11/16 (68.75%)	H&E, immunohistochemistry, immunofluorescence, therapy response evaluation and resistance mechanism exploration
[29]	CRC and GI cancers	Endoscopic biopsies and needle biopsies	110	70%	H&E, immunohistochemistry, next-generation sequencing, therapy response evaluation
[31]	EAC	Endoscopic biopsies	28	16/28 (57.2%)	H&E, immunohistochemistry, short tandem repeat analysis, whole-exome sequencing,

[i]ESCC: oesophageal squamous cell carcinoma; EAC: oesophageal adenocarcinoma cancer; CRC: colorectal cancer; GI: gastrointestinal; H&E: haematoxylin-eosin staining.

#### The establishment and application of PDX models in EC

Among reported studies establishing preclinical models of EC, PDX models derived from ESCC patients were more frequently observed than those derived from EAC patients (Table 3). A study established 18 EAC PDX models and confirmed their clinicopathological features [32]. A previous study established 4 ESCC and 13 EAC PDX models from a tumour tissue bank [33]. Zhu et al. reported a 61-PDX sequence, one of the largest ESCC PDX cohorts ever reported, and revealed that EGFR may function as a predictive biomarker for cetuximab response [34]. Dodbiba et al. established 21 PDX models from oesophageal/gastroesophageal junction cancers with a success rate of 38%, and among 7 xenografts with drug tests, only the chemosensitivity of 2 xenografts correlated with the patients’ clinical responses [35]. Zou et al. completed the modelling of 25 PDXs derived from 188 fresh endoscopic biopsy tissues of ESCC patients and established PDX models that retained the histologic and genomic characterizations. Tumour growth inhibition from 5 xenografts exposed to paclitaxel and platinum correlated well with the clinical response of patients [36]. Zhang et al. established 37 ESCC PDX models for preclinical drug discovery, in which models carrying HER2 expression had no response to 5-FU/cisplatin [37]. By applying a humanized ESCC PDX model, Liu et al. reported that indomethacin exerted antitumour activity and enhanced cancer immune responses [38]. Ma et al. analysed the heterogeneity of ESCC across 10 cell lines, 80 TCGA tissues and 2 PDX models at the DNA, RNA and protein levels and characterized various novel TP53 mutations, ECM-receptor interactions, focal adhesion, and olfactory transduction pathways (CNGB1) as indicators for accurate research and precision therapeutic development [39]. These observations highlight that EC PDXs has been established as a reliable preclinical model system with histology, genomic variation, and gene expression patterns consistent to the primary tumor, and have proved to be of great application value in oesophageal cancer drug screening.

**Table 3:** The establishment and application of PDX models in EC

Reference	Sample source	Tissue type	Sample number	Success rate of transplantation	Evaluation
[32]	EAC	Surgical resections	54	18/54 (33.3%)	H&E, short tandem repeat analysis
[33]	EAC/ESCC	Surgical resections, endoscopic biopsies and needle biopsies	61	17/61 (27.9%)	H&E, immunohistochemistry
[34]	ESCC	Surgical resections	110	61/110 (55.5%)	Immunohistochemistry, gene copy number and mutation analysis
[35]	EC/GEJ cancer	Surgical resections	55	21/55 (38.2%)	Immunohistochemistry
[36]	ESCC	Endoscopic biopsies	188	25/188 (13.3%)	Immunohistochemistry, DNA sequencing, therapy response evaluation
[37]	ESCC	Surgical resections	96	37/96 (38.5%)	Immunohistochemistry
[38]	ESCC	NA	3	NA	NA
[39]	ESCC	NA	2	NA	Proteomics analysis

[i]ESCC: oesophageal squamous cell carcinoma; EAC: oesophageal adenocarcinoma cancer; EC: oesophageal cancer; GEJ: gastroesophageal junction; H&E: haematoxylin-eosin staining; NA: not available

### Organoid and PDX models in GC

#### The culture and application of organoids in GC

PDOs of GC could be cultured from both tissue specimens and malignant ascites (Table 4) [40–43]. Gao et al. developed 15 GC PDOs from 5 patients and identified similar KRAS alterations and drug sensitivity in primary tumours and paired organoids [41]. Malignant ascites from advanced GC with peritoneal metastasis could be collected to generate organoids, showing divergence between individuals but comparability between ascites and PDOs in histological and genomic landscapes. Additionally, ascites-derived PDOs could be used to evaluate the response of chemotherapy regimens and showed similar drug sensitivity to that of patients [40]. Steele et al. reported that GC PDOs from individuals exhibited divergent morphological features and therapeutic regimen efficiency [42]. One patient exhibited a complete response clinically in accordance with the high sensitivity of their corresponding organoids, while another patient did not show a response to therapy agents even though the derived organoids partially responded. Overall, PDO culture techniques allow the generation of preclinical models from metastatic ascites or primary cancer sites, which representing the molecular characteristics and corresponding medical responses similar to parental tumour.

GC PDOs could be expanded long term and subjected to whole-genome sequencing, which can reveal the characteristic mutation style in specific subtypes of GC, such as *TP53* mutation in the CIN (chromosomal instability) group, *PIK3CA* alteration in EB virus, MSI (microsatellite instability), and GS (genomically stable) subtypes. Different types of *ERBB2* alterations including amplification and Ser310Phe showed similar regulatory patterns involving the c-MYC-mediated genes *CCND2*, *CDKN1A* and *THBS1* [44]. On the basis of biomarker detection, ex vivo targeted therapy tests were set up, showing that PDOs harbouring *HER2* mutations responded to trastuzumab alone or with 5-fluorouracil. The divergent response to classic chemotherapeutics was investigated, and the IC_50_ was compared with several cell lines showing a resistance tendency [45]. Therefore, PDO may be a powerful tool for the investigation of molecular pathogenesis and the discovery of biomarkers and targeted medicine.

GC organoid biobanks have been established and comprise an assortment of histological and molecular subtypes, and these normal and cancerous organoid lines recapitulate the morphological, histological, genetic, and transcriptomic characterization of corresponding tumour tissues [46]. Whole-exome sequencing in the GC PDOs revealed the well-documented driver mutations previously reported in GC, such as frequent alterations of *CDH1* in diffuse type, *TP53* in intestinal type and some other mutations involving *RHOA, ERBB2, FGFR2*, and *MYC*, and the similarities in the CIN and GS status to those previously reported in GC were also demonstrated. Organoid-based drug sensitivity ex vivo correlated well with clinical response. For instance, two patients who benefited from 5-fluorouracil and cisplatin after gastrectomy with this combined treatment had sensitive organoids, and another organoid derived from a patient with progressive disease showed no response to capecitabine. High-throughput drug screening was performed in PDOs from 7 patients, and the heterogeneity of agent response was assessed under the conditions of the same patient being given an array of drugs, the same drug being given to various individual patients or spatially different tumour regions from same patient being assessed [46]. Five characteristic organoids derived from gastroesophageal cancer patients in a gastrointestinal cancer cohort were established that captured the histological and genomic features of the parent tissues, such as the intestinal type, diffuse type, *ERBB2* amplification type, and temporal intratumor heterogeneity from the same patient (from baseline and posttreatment) [29]. The drug sensitivity of organoids correlated well with clinical treatment response, and the transformation of PDXs from sensitive to resistant to paclitaxel was sequentially generated before and after treatment [29].

Currently, immunotherapy has become a major therapeutic option in the clinic for most cancers, including gastroesophageal cancers. PDOs are being developed to explore the potential mechanisms of immune therapy resistance/response in gastroesophageal cancers by using coculture or air-liquid interface (ALI) systems [47–50]. A study presented a system for the coculture of mouse-derived gastric cancer organoids with immune cells, allowing the identification of a subgroup of gastric cancer patients who would potentially benefit from immunotherapy [49]. Chakrabarti et al. cocultured human gastric cancer organoids (huTGOs) generated from biopsied or resected tissues with cytotoxic T lymphocytes (CTLs) and myeloid-derived suppressor cells (MDSCs) and suggested that HER2-targeted therapy could inhibit CTL effector functions and PD-L1 expression [51]. In ALI system, tumor tissue containing stromal cells and immune cells are separated physically or enzymatically, following by seeded in the collagen gel in a upper surface which is exposed to air-conditions with a porous membrane underneath for nutrient diffusion occurring, so that oxygen can be transported in a more efficient manner [52]. By using ALI technology, Neal and colleagues successfully established co-culture PDOs containing immune cells or fibroblasts from 100 patients representing 28 different tumour types with the success rate of 73% after culture for one-month. These co-culture models maintained the diversity of T cell clones in patients for several weeks [50]. With the rapid development of organoid coculture technology, it provides an valuable platform for further research of personalized immunotherapy. However, the coculture PDOs still need to be more validated.

**Table 4:** Organoid culture and application in GC

Reference	Sample source	Tissue type	Sample number	Success rate of transplantation	Evaluation
[41]	GC	Surgical resections and endoscopic biopsies	15	14/15 (93.3%)	H&E, immunohistochemistry, immunofluorescence, whole-exome sequencing, next-generation sequencing, therapy response evaluation
[40]	GC	Malignant ascites	12	11/12 (91.7%)	H&E, immunohistochemistry, whole-exome sequencing, therapy response evaluation
[46]	GC	NA	63	> 90%	H&E, immunohistochemistry, whole-exome sequencing and transcriptome analysis, therapy response evaluation
[53]	GEP-NEN	Fresh clinical samples	16 GEP-NET and 22 NEC lines	NA	H&E, immunohistochemistry, whole-exome sequencing and transcriptome analysis

[i]GC: gastric cancer; GEJ: gastroesophageal junction; H&E: haematoxylin-eosin staining; NA: not available; GEP-NEN: gastroenteropancreatic neuroendocrine neoplasm; NEC: neuroendocrine carcinoma

#### The establishment and application of PDX models in GC

GC PDX models have been established so that the correlations compared to parent tumours, characterized by histology, genetics and clinical responses, can be evaluated (Table 5) [54–57]. Wang et al. constructed 9 PDX models from 32 GC patients (28.1% success rate) harbouring molecular heterogeneity, including HER2 positivity, *c-Met* overexpression, and *FGFR2* amplification, that responded to molecular targeted therapeutic agents [54]. Gastroscopic biopsies of GC patients were obtained to establish the PDX model, and the overall success rate was 34.1%, in which samples obtained before chemotherapy showed a higher transplantation rate. In addition to the concordance of histopathology and HER2 expression, chemosensitivity between parent tumour tissues and xenografts was investigated, revealing comparable therapeutic responses of corresponding regimens used in clinical treatment [55]. Within these cases, histological transformation from intestinal to diffuse type occurred in case 144, displaying no correlation between PDX-based drug sensitivity and clinically stable disease status [55]. Wang et al. developed mini PDX models for 4 GC patients to achieve personalized screening of chemotherapy or targeted therapy agents [58].

Notably, PDXs had become a successful tool for drug discovery in GC cancer. Ryan et al. constructed a comprehensive PDX collection of gastroesophageal cancer, including 46 (47%) GC adenocarcinomas, 25 gastroesophageal junction adenocarcinomas (26%), 21 oesophageal adenocarcinomas (32%), and three squamous cell carcinomas (3%), and then evaluated the antitumour activity of rational combination strategies [59]. Song et al. established patient-derived cell lines with peritoneal carcinomatosis, transformed them into orthotopic mouse models, identified major expression and activation traits, and then recapitulated the molecular and phenotypical features of donors [60]. Kuwata et al. successfully established 35 gastric cancer PDX models from 232 engrafted tissues and compared the clinicopathological factors associated with the establishment of PDX and CDX models [61]. Yagishita et al. built a large-scale Japanese patient-derived xenograft library (J-PDX) composed of 298 cross-cancer PDXs, in which 9 PDXs were gastric cancer, with a success rate of 16.7% (9/54) for engraftment [62]. Corso et al. established a comprehensive collection of gastric cancer preclinical models composed of 100 PDX and derivative cell lines or organoids, which included all the major gastric cancer histologic and molecular types identified by The Cancer Genome Atlas [63]. Chen et al. provided a 50-case PDX cohort of gastric cancer, characterized each of their individual histopathological and molecular features, and then evaluated anticancer agents targeting *MET, EGFR, HER2* and *CDKs* in these models [64]. The broad application of these PDX accelerate the development of individualized combination therapies and guide the design of future clinical trials.

**Table 5:** The establishment and application of PDX models in GC

Reference	Sample source	Tissue type	Sample number	Success rate of transplantation	Evaluation
[59]	GC/EC	Surgical resections, endoscopic biopsies and needle biopsies	276	98/276 (35.5%)	DNA sequencing, therapy response evaluation
[60]	GC	NA	3	NA	Karyotyping, whole-exome sequencing, RNA sequencing, and functional studies
[61]	GC	Surgical resections	232	35/232 (15.1%)	Immunohistochemistry
[62]	GC	Surgical resections, endoscopic biopsies, needle biopsies, pleural fluid and ascites	54	9/54 (16.7%)	Immunohistochemistry
[58]	GC	Endoscopic biopsies	4	NA	Therapy response evaluation
[54]	GC	Surgical resections	32	9/32 (28.1%)	Immunohistochemistry, fluorescent in situ hybridization, therapy response evaluation
[64]	GC	NA	50	NA	Immunohistochemistry, fluorescent in situ hybridization, next-generation sequencing, therapy response evaluation

[i]GC: gastric cancer; EC: oesophageal cancer; NA: not available

### Future prospects and challenges of PDO and PDX models in upper GI cancers

#### Future prospects and challenges of PDO

Precision medicine therapies generally require genomics based on targeted therapies and immunotherapies instead of “one-size-fits-all” chemotherapies [65]. Organoids recapture key characteristics of their corresponding normal or diseased organs and are amenable to current experimental technology. Inspired by cutting edge organoid technology, a deeper understanding of developmental biology and cancer biology could be achieved, as well as the filling of the gap between bench and bedside [19, 66–69]. Great progress has been accomplished in the establishment of PDO models and their application as a predictive preclinical model to evaluate therapeutic responses in vitro, involving lung cancer [70–72], colorectal cancer [29, 73–75], breast cancer [76, 77], ovarian cancer [78, 79], pancreatic cancer [80, 81], liver cancer [82, 83], neuroendocrine neoplasms [53], oesophageal cancer and gastric cancer. However, the following practical challenges still need to be addressed to completely maximize the potential value of upper gastrointestinal PDOs in predicting clinical response:

1. Optimization of the culture system. With regard to the flexible and diversified application in different scenarios such as with a large cohort for drug screening, omics sequencing (RNA sequencing, single-cell sequencing, etc.), and biobanking of organoids, sustainable expansion of patient-derived tumours and healthy organoids are greatly needed. However, the success rates were comparable for different types and subtypes of cancers. As shown in Tables 2 and 4, the success rate of organoid establishment in gastric cancer was more than 90%, which was significantly higher than that of esophageal cancer, which has a success rates ranging of 31.25-70%. Interestingly, in the existing research, EAC shows a lower success rate than ESCC, even if both of them originate from the esophagus. One of the reason may be different cancer need different culture condition. It has been indicated that multifarious protocols involving Wnt, epidermal growth factor, Noggin, and R-spondin1 showed dramatically divergent effects on organoid outgrowth. Establishment of a standard (basic) protocol for the main type of cancer is a prerequisite, and individualized adjustments based on the characteristics of tumour or healthy tissues are of great value. Except for inhibitors/agonists of specific pathways, the extracellular matrix has an important influence on the formation of organoids.
2. Quality control. The establishment of organoids depends on the complexity of resection or biopsy tissues or malignant ascites, which might comprise different contents, including cancer cells, normal epithelial cells, mesenchymal-derived cells, or immune cells. To some degree, the existence of varied types of cells reflects the complex tumour microenvironment, while the overexpansion of nonneoplastic portions would mislead the application of organoids in preclinical response to different therapeutic agents. A balance between the presence or absence of normal tissues should be defined to improve the precision of organoids in modelling and clinical prediction.
3. Drug sensitivity evaluation. The clinical application of organoids has been well developed, either in retrospective observation studies of the consistency between clinical responses and organoid-based drug sensitivity or in intervention studies to predict the individual probable efficacy of potential therapies. Several evaluation indices have been introduced to evaluate the efficiency of different anticancer drugs, including image-based indices such as the size, area, and deep neural network-developed quantification using bright field- or fluorescent field-derived images or cell viability-based indices such as ATP-based AUC or IC_50_ and flow cytometry-based cell viability. Multidimensional and diversified evaluation approaches should be developed to diagnose drug-induced cell viability to further analyse the relationship between sensitivity results and treatment outcomes. Furthermore, the inhibitors and growth factors in medium may affect gene expression and signal pathway of tumour arganoids, thereby have effects on drug sensitivity.
4. Microenvironment remodelling. Organoids have been established that can reproduce the genomic landscape and structure of parental tissues, but not all aspects of the microenvironment have been recapitulated, such as mesenchymal-derived cells or immune cells. The preservation of immune cells has been found in ALI-derived organoids, at least for a short term of a month, as mentioned above [50]. However, unlike epithelial cell, which can be continuously passaged and cryoppreserved, the immune element of ALI PDO (such as TILs, TAMs and fibroblast stroma) declines over time and are difficult to persist beyond 60 days [50]. Further optimization of the coculture system is necessary in the future. Besides, since the peripheral immune system play a critical role in antitumour immunity, coculture of organoids with immune components from lymph nodes or peripheral blood may create more comprehensive models that mimic the microenvironment of patients. A coculture system involving tumour organoids and T-cell populations derived from either peripheral blood lymphocytes or TILs was used to explore their potential application in practical immune checkpoint inhibitor testing, which was further adapted into cell therapy evaluations, such as CAR-T cells. In the future, improving the practicality remains an issue to broaden the utility of organoids in preclinical prediction.
5. Large cohorts with paired organoid-related information and clinical follow-up. Efforts have been invested to probe the possibility of using organoids as biomarkers to predict the response to potential regimens. Among a wealth of clinical studies with organoid-based drug sensitivity tests, a vast number of tumour organoids have been established, while censored data either in drug screening or clinical responses have been found, leading to limited data to determine the potential correlation between organoid-derived drug testing results and clinical responses. Prospective and integral cohort studies are urgently needed to provide valuable insights into individualized precision medicine.

#### Future prospects and challenges of PDX

As we all known, the PDX is widely used in basic research, drug development and clinical practice because it shows significant heterogeneity of primary tumour and is highly consistent with patient response to treatment. Nevertheless, there are still some limitations of PDX models in cancer research:

1. Time course. The establishment of PDX is time consuming, which is a major limiting factor in the application of PDX models in real-time personalized medicine. In the literature, establishment of PDX model usually takes 4–8 months, much longer than clinically acceptable waiting times to start treatment.
2. Engraftment rate. The successful rate of developing PDX models varies among tumor types. As described above, the PDX model has a success rate of 13.3-55.5% and 15.1-35.5% in EC and GC, respectively. There is an urgent need to further optimize the PDX culture technology for various tumors.
3. Microenvironment remodelling. Human tumor stromal cells and extracellular matrix are gradually replaced by murine counterparts after transplanting into immunodeficient mice [84], and the exact effect of these murine stroma in PDX models remains unclear. Besides, immunodeficient mice lacks components of human immune system, which makes PDX models difficult to study the tumor immune environment and develop immunotherapy strategies. Fortunately, humanized mice, which are immunodeficient mice co-engrafted with human tumours and human hematopoietic stem cells or immune components to reconstitute human immune system, are being investigated [85]. These humanized PDX model offers a potential platform for studying immunotherapies, despite the lack of HLA molecule and lesser functionality of immune cells.

## Data Availability

All the information generated and analysed is included in the manuscript and relevant tables and figures. Since this is a systematic review of the available literature, no raw data were collected.

## Abbreviations

PDO: patient-derived organoid
PDX: patient-derived xenograft
GI: gastrointestinal
GC: gastric cancer
EC: oesophageal cancer
ESCC: oesophageal squamous cell carcinoma
EAC: oesophageal adenocarcinoma cancer
CRC: colorectal cancer
NGS: next-generation sequencing
CDX: cell line-derived xenograft
E-MEOM: oesophageal minimum essential organoid culture medium
BE: Barrett’s oesophagus
STR: short tandem repeat
WES: whole-exome sequencing
CIN: chromosomal instability
MSI: microsatellite instability
GS: genomically stable
ALI: air-liquid interface
CTLs: cytotoxic T-lymphocytes
MDSCs: myeloid-derived suppressor cells
GEP-NEN: gastroenteropancreatic neuroendocrine neoplasm
NEC: neuroendocrine carcinoma
